# Development of a Core Patient-Reported Outcome (Measures) Set for Pediatric Physical Therapy

**DOI:** 10.1097/PEP.0000000000001304

**Published:** 2026-07-31

**Authors:** Selina Limmen, Dorinde L. Korteling, Manon A. T. Bloemen, Michiel A. J. Luijten, Raoul H. H. Engelbert, Eugene A. A. Rameckers, Hedy A. van Oers, Lotte Haverman, Marjolijn Ketelaar

**Affiliations:** Department of Child and Adolescent Psychiatry & Psychosocial Care, Amsterdam UMC, University of Amsterdam, Emma Children’s Hospital, Amsterdam, The Netherlands (Limmen, Korteling, Luijten, van Oers, and Haverman); Child Development, Amsterdam Reproduction and Development, Amsterdam, The Netherlands (Limmen, Korteling, Luijten, van Oers, and Haverman); Mental Health, Amsterdam Public Health, Amsterdam (Limmen, Korteling, Luijten, van Oers, and Haverman); Personalized Medicine, Amsterdam Public Health, Amsterdam (Limmen); Methodology, Amsterdam Public Health, Amsterdam (Korteling, Luijten); Research Group Moving, Growing, and Thriving Together, HU University of Applied Sciences Utrecht, Utrecht, The Netherlands (Bloemen); Department of Epidemiology and Data Science, Amsterdam UMC, Vrije Universiteit, Amsterdam, The Netherlands (Luijten); Department of Rehabilitation Medicine, Amsterdam Movement Sciences, Amsterdam UMC, University of Amsterdam, Amsterdam, The Netherlands (Engelbert); Centre of Expertise Urban Vitality, Faculty of Health, Amsterdam University of Applied Sciences, Amsterdam, The Netherlands (Engelbert); CAPHRI, Maastricht University, Maastricht, The Netherlands (Rameckers); Center of Expertise, Adelante Rehabilitation, Valkenburg, The Netherlands (Rameckers); Rehabilitation Science and Physiotherapy, REVAL, Hasselt University, Hasselt, Belgium (Rameckers); Amsterdam Public Health, Quality of Care, Amsterdam, The Netherlands (van Oers); Digital Health, Amsterdam Public Health, Amsterdam, The Netherlands (Haverman); UMC Utrecht Brain Center, University Medical Center Utrecht, Utrecht, The Netherlands (Ketelaar); and De Hoogstraat Rehabilitation, Center of Excellence for Rehabilitation Medicine Utrecht, Utrecht, The Netherlands (Ketelaar).

**Keywords:** patient-reported outcome measures, pediatric physical therapy, patient-centered care, patient-centered research, pediatric rehabilitation, quality of life

## Abstract

**Purpose:**

To develop a core set of relevant patient-reported outcomes (PROs) and corresponding measures (PROMs) for pediatric physical therapy, suitable for daily practice, research, and quality assessment.

**Methods:**

The research group defined the core set’s scope. Relevant PROs were identified through focus groups and interviews with adolescents, parents, and pediatric physical therapists, and supplemented by a scoping review. Findings were assessed by an expert panel. PROs were prioritized via a survey among pediatric physical therapists and selected for the core set. Valid PROMs were selected for each PRO.

**Results:**

Nine PROs were selected, grouped into 3 domains: functioning (physical functioning, participation, social functioning), symptoms (pain and fatigue intensity/interference), and overarching (quality of life, perceived overall health). Suitable PROMs were identified for all except “participation.”

**Conclusions:**

A core PRO(M) set for pediatric physical therapy is presented, with recommendations for use and implementation. Further research should develop a feasible generic PROM for participation.

**WHAT THIS EVIDENCE ADDS**

Current evidence: Patient-reported outcomes measures (PROMs) provide insight into patient perceptions of health, functioning, and quality of life,^1^ and support patient-centered care, communication, and treatment evaluation.^2,3^ Previous research recognized the importance of PROMs in daily pediatric physical therapy (PPT) practice, research, and quality assessments, but stated difficulties in selecting valid and reliable PROMs.^4,5^ Significant challenges in their use include inconsistent terminology of the outcomes^6^ and extensive variety of PROMs.^7^ This vast array of PROMs complicates the selection of internationally recognized, valid, and reliable measures for clinical practice and research.

Gap in the evidence: Insight into relevant patient-reported outcomes (PROs) and standardization of PROMs in PPT is necessary to provide guidance on PROM use in this field. As such, this study aimed to develop a generic core PRO(M) set for children aged 5 to 18 years in PPT, applicable in clinical practice, research, and quality assessments.

How does this study fill this evidence gap? This study developed the first consensus-based, generic core PRO(M) set, relevant for all children within PPT, and suitable for daily practice, research, and quality assessment. The resulting set includes 9 core PROs across functioning, symptoms, and overarching domains, with recommended PROMs for each outcome (except participation, where no suitable PROM was found).

Implication of all the evidence: This study demonstrates that a selection of generic PROs are universally relevant to all children, regardless of their (medical) condition. The core PROM set could be supplemented with disease/condition-specific PROMs when necessary. Additionally, this study highlights the need for future research to develop a feasible PROM for participation, sensitive to the nuanced nature of this construct.

## INTRODUCTION

Pediatric physical therapy (PPT) addresses movement challenges in children, such as congenital and developmental disorders, with interventions tailored to personalized goals that promote independence and participation in daily life.^8,9^ Alongside general physical therapy, PPT includes a holistic approach, integrating physical, social, and mental well-being into healthcare within the family dynamics and a pedagogical approach.^8,9^ This comprehensive perspective ensures that treatment addresses the multidimensional aspects of a child’s health by promoting physical, mental, and social development and quality of life.

Patient-reported outcomes (PROs) provide insight into patient perceptions of health, functioning, and quality of life.^1^ PROs are directly reported by the patient or a proxy (such as a parent/caregiver), without the involvement of a healthcare professional, and are measured using valid and reliable patient-reported outcome measures (PROMs).^1^ PROMs can be used in clinical practice, research, and quality assessments.^10,11^ Efficacy studies have shown that PROM use in clinical practice promotes personalized care, patient-provider communication, and consultation preparation.^2,3^ Alongside other outcome measures, PROMs can play an important role in guiding the pediatric physical therapist’s clinical reasoning by providing structured insights into the patient’s functioning, needs, and treatment progress from the perspective of the child and their parents. PROMs can contribute to shared decision-making^12^ and facilitate goal setting^13^ by identifying key concerns and functional limitations from a patient or parent perspective. Within research, PROMs allow for systematic incorporation of the patient voice, guiding the development of interventions positively impacting patients’ experiences.^14^

Despite the recognition of the importance of PROMs in daily PPT practice, research, and care quality assessments there are noted difficulties in selecting valid and reliable PROMs for PPT.^4,5^ Previous studies suggest that one contributing factor is the inconsistent terminology used to describe PROs, along with the absence of a clear definition. For example, various outcome sets use terms such as “activities of daily living,” “activity limitations,” “disability,” “functional status,” “mobility,” and “motor function,” although it remains unclear whether these terms represent distinct constructs or refer to the same underlying concept, such as “physical function(ing).”^6^ The use of varied terminology for the same PRO complicates comparisons and standardization within and across diagnoses, reducing clarity and interpretability of intervention studies.^6^ Another issue highlighted in previous research, both within and outside PPT, is the extensive variety of PROMs utilized. In a scoping review on PROs in PPT intervention studies, 158 PROMs were used to measure 40 PROs.^7^ This large number of PROMs complicates the selection of internationally recognized, valid, and reliable measures.

To better understand these issues, it is essential to distinguish between disease-specific and generic PROMs. Disease-specific PROMs are designed to assess PROs relevant to a particular patient group with the same medical condition, whereas generic PROMs capture PROs relevant across diverse patient populations.^15^ It is often assumed that different PROMs are required for specific patient groups, a belief that may be present within PPT due to the heterogeneous nature of the patient population in terms of age, condition, and level of functioning. However, content analyses of disease-specific PROMs show that they often contain PROs relevant across various patient groups.^6,16,17^ Using disease-specific PROMs to measure the same underlying PRO impedes data aggregation across patient populations, as different PROMs often use different scoring systems. Additionally, it also complicates PROM use, as different PROMs are selected and interpreted for each medical condition. Furthermore, in light of sustainability and feasibility, continued use and maintenance of numerous disease-specific PROMs is unrealistic. Therefore, generic PROMs are preferred, supplemented with disease-specific measures when clinically relevant.^16,18^ To provide guidance on PROM use in PPT, insight into relevant PROs and standardization of PROMs in this field is necessary. As such, we aim to develop a generic core PRO(M) set for children aged 5–18 years in PPT, applicable in clinical practice, research, and quality assessments.

## METHODS

**Fig. 1.**
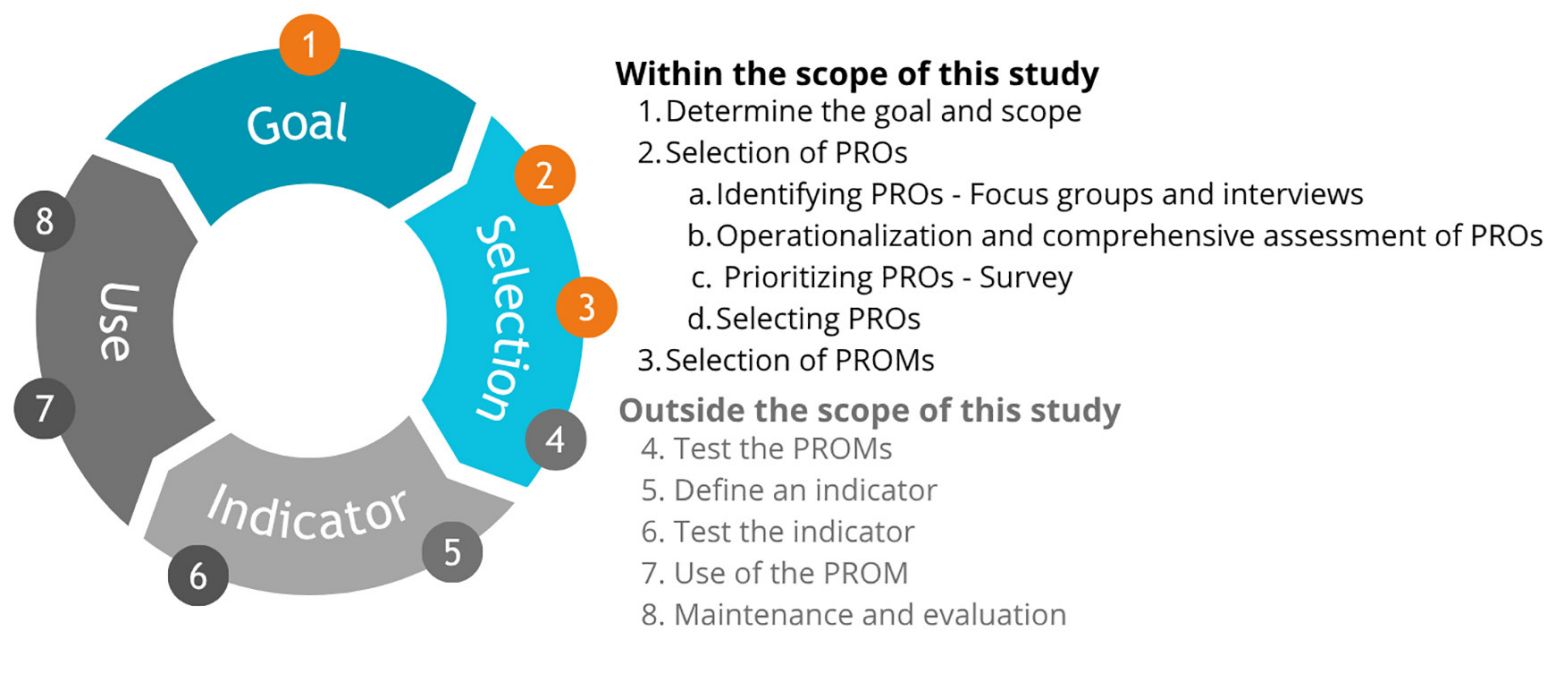
The PROM cycle. Adapted from van der Wees et al^19^ with permission. Its application is illustrated by Oude Voshaar et al.^20^ PROM, patient-reported outcomes measures.

This exploratory sequential study followed the first 3 steps of the PROM-cycle (Figure 1),^19^ a framework for selecting and implementing PROMs. This study is part of the larger project *Shared Decision-Making in Pediatric Physical Therapy*. Throughout several phases of the current study, the project’s research group served as an expert panel. This group consisted of researchers experienced in PPT (n = 4), psychology, PROMs and psychometrics (n = 4), and rehabilitation and patient involvement (n = 1). One member was affiliated with the Dutch Association of Pediatric Physical Therapy (Nederlandse Vereniging voor Kinderfysiotherapie), and 5 members with the KLIK PROM expertise center at Amsterdam University Medical Center (UMC).^21^ This study was reported in accordance with the Core Outcome Set-STAndards for Reporting (COS-STAR) guideline for studies developing a core outcome set.^22^ It should be noted that this study focuses solely on the development of a core PROM set rather than a full core outcome set (which would include additional outcome types such as clinical measures). Ethical approval for this study has been granted by the Medical Ethics Review Committee of the Amsterdam UMC (W23_005 # 23.026).

### Step 1: Determine goal and scope

In a live meeting, the goal and scope of the core PRO(M) set was determined by the expert panel.

### Step 2: Selection and operationalization of PROs

#### Step 2a: Identifying PROs—focus groups and interviews

##### Participants.

Focus groups and individual interviews were conducted with (1) adolescents aged 12–18 years receiving PPT, (2) parents of children aged 5–18 years receiving PPT, and (3) registered pediatric physical therapists. Participants were recruited through PPT practices in primary healthcare in the Netherlands. Purposive sampling (including a variety of ages, conditions, and levels of physical functioning) supplemented with convenience sampling was used. All participants provided written consent. Participants received a €15 gift card.

##### Data collection.

Separate focus groups, including 3 to 8 people, were conducted for each participant group. Focus groups with adolescents and parents were conducted in person at various PPT practices throughout the Netherlands whenever feasible. If in-person focus groups were not feasible, video chat (Microsoft Teams) was used. Participants could opt for an individual interview via videochat if preferred. The audio of all focus groups and individual interviews was recorded. Focus groups lasted 120 minutes and were moderated by a researcher with experience in qualitative research and/or PPT. A co-moderator was present at each discussion. Individual interviews lasted approximately 60 minutes and were conducted by 1 researcher. The focus groups and interviews consisted of 2 parts:

###### Part 1

The first part focused on the impact of a child’s medical condition on daily life. During focus groups with adolescents, a pediatric patient engagement game (All Voices Count^23^) was used. For parents and PPTs, the “Complain and Cheer wall”^24^ technique was used.

###### Part 2

The moderator and co-moderator gathered all the PRO-constructs provided by participants. Subsequently, participants selected and ranked relevant PRO constructs to measure in PPT. A discussion followed to explore the identified themes.

##### Analysis focus groups and interviews.

Audio recordings were transcribed verbatim using “Microsoft WordOnline” for in-person sessions and “Microsoft Teams” for online sessions, after which transcripts were manually corrected. All transcripts were pseudonymized and sociodemographic information was secured in a coded subject identification log. Results were analyzed by 2 researchers using thematic analysis within a deductive grounded theory approach, utilizing MaxQDA. First, text fragments were open-coded into data-driven concepts. 25% of transcripts were independently open-coded by 2 researchers, after which work was compared until consensus was reached. The remaining 75% of transcripts were divided equally. Second, open codes were linked by 2 researchers to preexisting health domains and operationalizations from the “Advisory Report Set of Generic PRO(M)s for Children” (GPROM set),^25^ an initiative of the Dutch Ministry of Health, Welfare and Sport. This report recommends a standard set of PROs and generic PROMs for children, based on the adult standard set by Oude Voshaar et al^20^ (Supplemental Digital Content 1, available at: https://links.lww.com/PPT/A696). The GPROM set includes PROs covering physical, mental, and social functioning as well as symptoms and overarching concepts. PROs not fitting into the GPROM set were categorized based on their description in the focus groups and interviews.

#### Step 2b: Operationalization and comprehensive assessment of PROs

We supplemented the focus groups/interviews with results from a previously published scoping review, which provided an overview of PROs measured in PPT.^7^ Outcomes from the literature search were operationalized by 2 researchers using the GPROM set as a guideline. Subsequently, operationalizations of both focus groups/interviews and the literature search were discussed by the expert panel until full consensus was reached. Operationalization of PROs was considered essential for the later selection of PROMs covering the intended concepts and to enable consistent use of PRO terminology across research and clinical practice.

In a separate meeting, the expert panel assessed which PROs to include in the prioritization using a stepwise process. The first 3 steps were performed separately for the PROs of the focus groups/interviews and the literature review. In step 1, non-PROs were removed. Outcomes were considered PROs if they pertained to the patient’s perception of health, functioning and/or quality of life and could be directly reported by a patient without interpretation of a healthcare professional. In step 2, PROs relevant for most children in PPT were identified, and disease-specific outcomes such as “asthma symptoms,” were removed. In step 3, the expert panel identified overlapping constructs and merged PROs with the same operationalization. In step 4, the remaining PROs from the focus groups/interviews, and review were compared and overlapping PROs were merged.

#### Step 2c: Prioritizing PROs—survey

##### Participants.

Survey participants were pediatric physical therapists from primary, secondary, and tertiary healthcare in the Netherlands. They were recruited through PPT practices, personal networks, and an announcement on the Dutch association for PPT platform.

##### Data collection.

Survey participants were presented with the PROs and corresponding operationalizations defined in step 2b. Each PRO was rated on a 5-point Likert scale, ranging from “Not at all important” to “Very important.” Surveys were administered through Microsoft Forms.

##### Analysis of survey

Survey results were analyzed using reweighted priority-setting,^26^ where a ranked list of themes is generated.

#### Step 2d: Selecting PROs

Based on the prioritization results (step 2c) and expert panel input, the set was divided into a core PRO set for all children in PPT and optional PROs relevant for some children in PPT.

### Step 3: Selection of PROMs

PROMs were selected to measure the PROs from the core PRO set. The expert panel decided to include unidimensional PRO constructs in the core PRO set to enable separate scoring of each PRO, thereby improving the interpretability of the PROM.^27^ Therefore, PROMs combining multiple domains into a single score (such as the Pediatric Quality of Life Inventory^28^) were excluded from consideration. PROMs were primarily selected from the GPROM set.^25^ During GPROM set development, a working group reviewed over 300 PROMs for content validity, usability, and psychometric properties. The resulting GPROM set predominantly includes Patient-Reported Outcomes Measurement Information System (PROMIS) instruments due to their generic nature, suitability across a broad age range (5–18 years), sufficient psychometric properties, and support of short forms and computerized adaptive testing (CAT).^14^ CATs reduce the number of questions asked by dynamically selecting the most informative items based on a respondent’s previous answers, thereby increasing efficiency without compromising measurement precision. To support standardized PROM use and data comparability, PROMIS instruments are available in multiple languages^14^ and ongoing initiatives are developing crosswalks to convert scores from other PROMs to PROMIS metrics.^14^ PROMIS also enables longitudinal monitoring across the lifespan with both pediatric and adult measures.^14^

If our core PRO set included a PRO not represented in the GPROM set,^25^ the expert panel selected a PROM based on usability and psychometric properties. For usability, we considered: (1) the comprehensiveness of the questions, (2) applicability across a wide age range (preferably 5–18 years), (3) a completion time of less than approximately 2.5 minutes (<12 items) per PROM,^28^ (4) no associated costs, and (5) available in multiple languages to support international use. For psychometric properties, we consulted the COnsensus-based COnsensus-based Standards for the selection of health Measurement Instruments (COSMIN) criteria for good measurement properties.^29^ Potential PROMs were found primarily via the PROMIS Health Organization (as recommended by the GPROM set^25^), and then through a scoping review on PRO(M)s in PPT, personal networks, and a website overviewing healthcare measurement instruments available in Dutch (www.meetinstrumentenzorg.nl). The expert panel formulated recommendations of use regarding the resulting PRO(M) set, based on prior knowledge in the fields of PPT and PROM implementation.

## RESULTS

### Step 1: Determine goal and scope

The expert panel determined that the generic core PRO(M)s set should be suitable for daily care, research, and quality assessment for all children aged 5–18 in PPT. Two key concepts were defined before data collection: the core PRO set should include only a limited number of PROs relevant to most children in PPT, and the core PROM set should be minimally burdensome, taking no more than about 10 minutes to complete.

### Step 2: Selection and operationalization of PROs

#### Step 2a: Identifying PROs—focus groups and interviews

Twelve focus groups/interviews were held between March and August 2023 with a total of 17 adolescents (3 focus groups; 2 individual interviews), 16 parents (4 focus groups; 1 individual interview), and 10 pediatric physical therapists (2 focus groups). Three out of 4 focus groups with parents, and all focus groups with PPTs, were held via video chat due to the geographically dispersed nature of participants. Sociodemographic information of the participants can be found in Table 1. Thirty-two PROs were identified in the first part of the focus groups/interviews, highlighting the impact of the condition on the child’s life, of which 20 were considered important for measurement in the PPT by participants in the second part of the focus groups/interviews. The scoping review identified 40 PROs.^7^ A list of all identified PROs is added in Supplemental Digital Content 2, available at: https://links.lww.com/PPT/A697.

**TABLE 1:** Sociodemographic Information of Focus Groups/Interviews and Survey Participants

Sociodemographic Information	Focus Groups/Interviews			Survey
	Adolescents (n = 16)	Parents (n = 16)	Pediatric Physical Therapists (n = 10)	Pediatric Physical Therapists (n = 95)
Age in years				
Mean [SD]	14.6 [2.0]	42.8 [5.2]	47.5 [10.6]	44.4 [11.8]
Range	11–18	32–52	35–63	25–71
Gender				
Male	6 (37.5%)	0	1 (10.0%)	13 (13.5%)
Female	10 (62.5%)	16 (100.0%)	9 (90.0%)	80 (83.3%)
Other/prefer not to say	0	0	0	2 (2.1%)
Highest educational level^a^				
Lower secondary	1 (6.3%)	1 (6.3%)	0	0
Upper secondary	14 (87.5%)	1 (6.3%)	0	0
Postsecondary nontertiary	1 (6.3%)	3 (18.8%)	0	0
Bachelor	0	10 (62.5%)	0	0
Master or doctoral	0	0	10 (100%)	95 (100%)
Missing	0	1 (6.3%)	0	0
Duration of PPT intervention (of child) in months				
Mean [SD]	40.9 [41.6]	48.2 [49.1]	/	/
Range	1–144	4–132	/	/
Medical condition/diagnoses of child^b^				
Cardiovascular and/or pulmonary [acute/chronic]	2 [0/2] (12.5%)	2 [0/2] (12.5%)	/	/
Musculoskeletal [acute/chronic]	12 [7/5] (75.0%)	3 [3/0] (18.8%)	/	/
Neurological [acute/chronic]	2 [0/2] (12.5%)	5 [0/5] (31.3%)	/	/
Combined [acute/chronic]	0	5 [0/5] (31.3%)^c^	/	/
Unknown	0	1 (6.3%)		
Work experience as a PPT in years				
Mean [SD]	/	/	22.6 [10.3]	17.4 [10.8]
Range	/	/	10–38	1–42
Care setting in which PPT has work experience^d^				
Primary care setting	/	/	9 (90.0%)	61 (63.5%)
Secondary care setting	/	/	6 (60.0%)	20 (20.8%)
Tertiary care setting	/	/	4 (40.0%)	16 (16.7%)

Abbreviation: PPT, Pediatric physical therapist.

a Parents and PPTs highest and completed level of education. Adolescents’ current education (categorized according to the International Standard Classification of Education (ISCED) levels^30^).

b Medical conditions or diagnoses were classified as chronic if their duration exceeded 3 months.

c These medical conditions/diagnoses include 2 unknown genetic syndromes, Gillespie syndrome, Ehlers-Danlos syndrome and Down syndrome.

d In the Dutch healthcare system, primary care comprises community‐based services (eg, general practitioners and physical therapists) that patients can access directly via self‐referral without a prior physician’s referral. Secondary care comprises medical specialist services in hospitals to which patients are referred by their general practitioner (including physical therapy in specialist settings) that generally require referral, whereas tertiary care denotes highly specialized services (eg academic medical center rehabilitation) for complex cases or advanced conditions.

#### Step 2b: Operationalization and comprehensive assessment of PROs

**Fig. 2.**
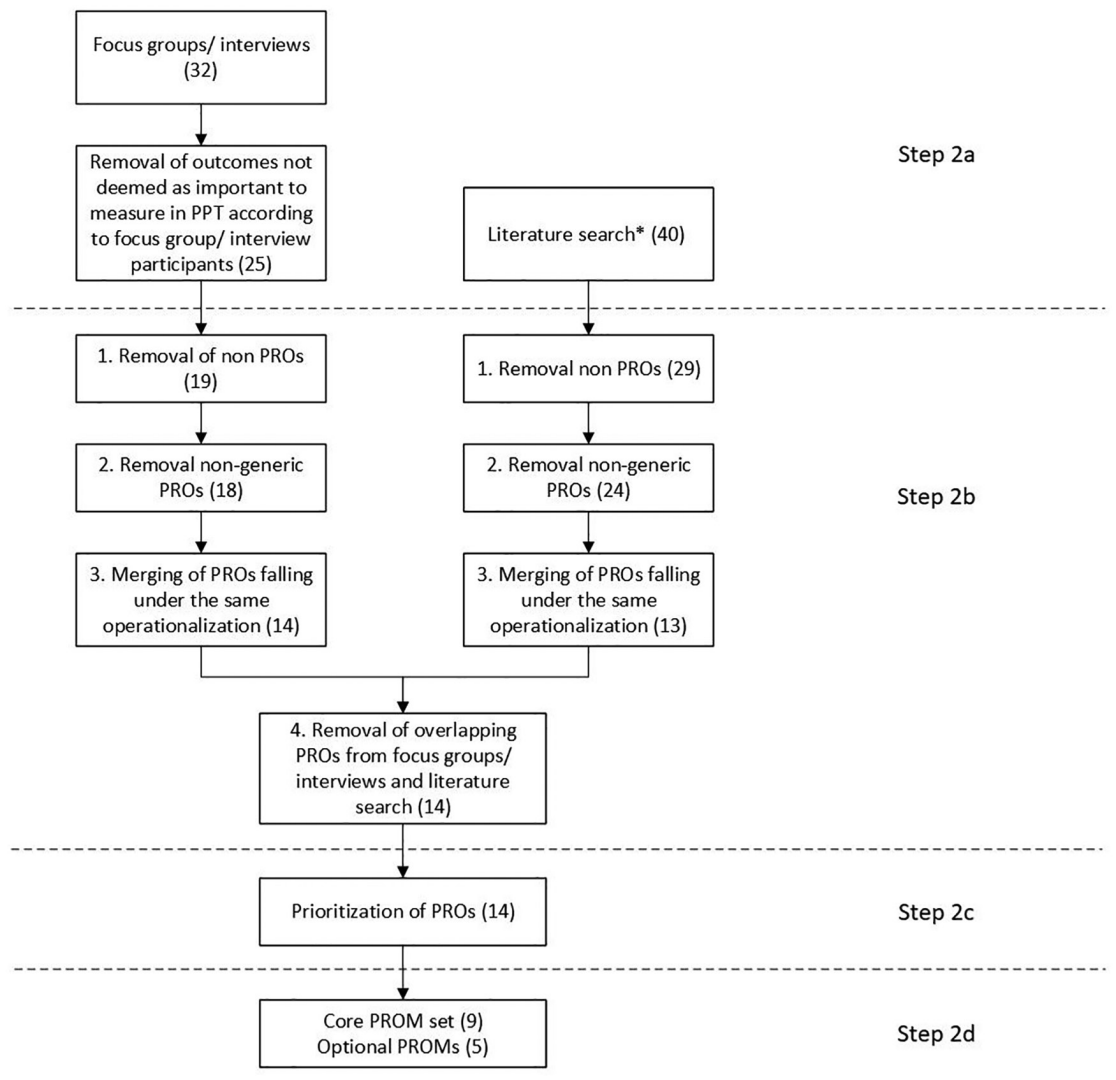
Flowchart of PRO assessment by expert panel (Step 2 of the PROM cycle). The number of PROs remaining is denoted in the brackets. *The literature review refers to the review performed by Korteling and Limmen et al.^7^ PRO, patient-reported outcome; PROM, patient-reported outcomes measures.

In February 2024, the expert panel operationalized the PROs from the literature review, and for a comprehensive assessment to determine which PROs to include in the prioritization phase. The expert panel removed (1) outcomes not classified as a PRO, (2) disease-specific PROs, (3) PROs falling under the same operationalization, and (4) overlapping PROs from the focus groups/interviews and literature review. This resulted in the prioritization of 14 PROs. Figure 2 shows a flowchart of the selection process. Supplemental Digital Content 2, available at: https://links.lww.com/PPT/A697, provides information on removal and operationalization.

#### Step 2c: Prioritizing PROs—survey

Between February and March 2024, 95 pediatric physical therapists completed the survey. Participant sociodemographic details are presented in Table 1. “Participation” was ranked as the most important PRO, followed by “physical functioning/activity.” Lowest ranked PROs were “anxiety,” “depression,” “anger,” and “sleep.”

#### Step 2d: Selecting PROs

**Fig. 3.**
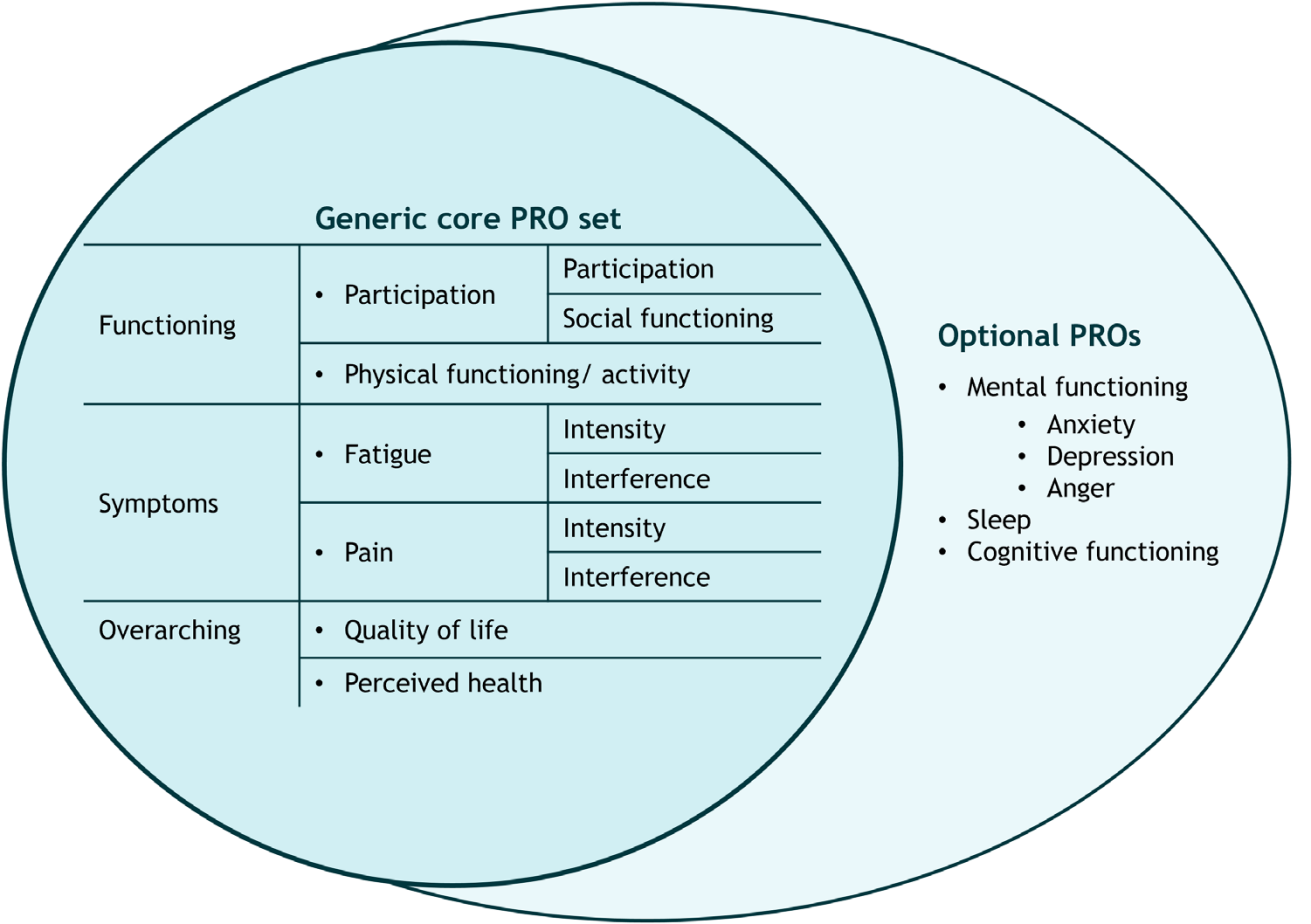
Generic core PRO set for use in pediatric physical therapy. PRO, patient-reported outcome.

After prioritization, the expert panel came together in March 2024 to formulate the generic core PRO set for PPT. The set was divided into the core PRO set, containing 9 PROs, and the optional PROs, containing 5 PROs (Figure 3). Although the PRO “cognitive functioning” was initially deemed non-generic in step 2b, the expert panel concluded in March 2024 that it is relevant to a broad group of children receiving PPT and not inherently condition- or disease-specific (and therefore generic in nature). Therefore, it was included as an optional PRO in the core PRO set.

### Step 3: Selection of PROMs

For the 9 core PROs, 6 suitable PROMs were selected from the GPROM set (Supplemental Digital Content 3, available at: https://links.lww.com/PPT/A698). For the 5 optional PROs, 4 PROMs were chosen from the GPROM set. For the PRO “physical functioning,” the GPROM set recommends the PROMIS pediatric Mobility short-form. However, as this PROM only addresses mobility and omits fine motor and upper extremity skills, we sought an alternative or additional PROM to ensure full coverage. In total, 3 core PROs (physical functioning/activity, participation, pain interference) and 1 optional PRO (cognitive functioning) remained without selected PROM after consulting the GPROM set (Supplemental Digital Content 3, available at: https://links.lww.com/PPT/A698). For these 4 PROs, 3 suitable PROMs were identified through the PROMIS Health Organization. Previously reported psychometric properties demonstrated sufficient psychometric quality for use in PPT (Supplemental Digital Content 3, available at: https://links.lww.com/PPT/A698). No suitable PROM was found for the PRO “participation.” One PROM was identified through PROMIS Health Organization, however this PROM showed insufficient usability as it was meant for adults. Another 6 potentially suitable PROMs fitting our operationalization were identified through the scoping review on PRO(M)s in PPT,^7^ personal networks and www.meetinstrumentenzorg.nl, 1 of which was not generic in nature, and 5 of which showed insufficient usability (Supplemental Digital Content 3, available at: https://links.lww.com/PPT/A698). The core PRO(M) set is presented in Table 2. The recommendation of use regarding the core PRO(M) set can be found in Table 3.

**TABLE 2:** Generic Core PRO(M) Set for Use in Pediatric Physical Therapy

Generic Core PRO(M) Set						
Generic PRO Concept					PROM/Proxy PROM	Items
Functioning	Participation			Participation	No suitable Pediatric/Proxy PROM found	
				Social functioning	PROMIS Pediatric/Parent Proxy Peer Relations CAT or SF4	4–12^a^
	Physical functioning/activities				PROMIS Pediatric/Parent Proxy Mobility CAT or SF4 and/or PROMIS Pediatric/Parent Proxy Upper Extremity CAT or SF4	4–12^a^ 4–12^a^
Symptoms	Fatigue		Intensity^b^		NRS Fatigue/Parent Proxy NRS Fatigue	1
			Interference		PROMIS Pediatric/Parent Proxy Fatigue SF4	4
	Pain		Intensity^b^		NRS Pain/Parent Proxy NRS Pain	1
			Interference		PROMIS Pediatric/Parent Proxy Pain Interference SF4	4
Overarching	Quality of life				NRS quality of life/Parent Proxy NRS quality of life	1
	Perceived health				NRS perceived health/Parent Proxy NRS perceived health	1
						Total: 12–48
**Optional PRO(M) Set**						
** Generic PRO Concept**					**PROM/Proxy PROM**	** Items**
Mental functioning		Anxiety			PROMIS CAT Pediatric/Parent Proxy Anxiety CAT or SF4	4-12^a^
		Depression			PROMIS CAT Pediatric/Parent Proxy Depression CAT or SF4	4–12^a^
		Anger			PROMIS Pediatric/Parent Proxy Anger SF5a	5
Sleep					PROMIS Pediatric/Parent Proxy Sleep disturbance SF4	4
Cognitive functioning					PROMIS Pediatric/Parent Proxy Cognitive function SF7	7

Abbrevuations: PRO, patient-reported outcomes; PROM, patient-reported outcomes measures; PROMIS, patient-reported outcomes measurement information system; SF4, 4 item short form; SF5, 5 item short form; SF7, 7 item short form.

a Range of 4–12 items for PROMIS Computer Adaptive Testing (CAT).

b NRS can be used as screening question for administering an interference PROM.

**TABLE 3:** Recommendations for Use

PROMs
• For PROMIS measures in the core PROM set, we recommend using the CAT version of these PROMs, due to their higher reliability and increased relative information gathered per question.^31,32^ However, administering CATs requires a link to the PROMIS assessment center, and there may be associated costs for each CAT administered. ○ If CAT use is not possible, PROMIS short forms are a good alternative. These are free of charge, generate comparable scores on the same scoring metric, and have shown sufficient psychometric properties.^15,32^
• To increase relevance of the core PRO(M) set for patients, it is possible to introduce flexibility within the set. ○ Depending on type of complaint/diagnosis, either PROMIS Mobility, PROMIS Upper Extremity, or both can be administered. ○ For symptoms, the NRS can be used as a screener for administering PROMs about interference to reduce the burden on the patient. ○ The core PROM set could be supplemented with disease/condition-specific PROMs, such as PROMIS Pediatric Asthma symptoms, when necessary.^15^
• Healthcare providers and researchers should evaluate whether the optional PROs are relevant for an individual patient or, in research settings, for a specific patient population.
Patients
• To preserve the intended unbiased answers on the PROMs, we recommend patients (and their parents) to complete the PROMs without the presence of a healthcare professional or researcher, for example at home.
• When possible, we recommend children completing the PROMs themselves, and using proxy PROMs only if the child is younger than 8 years or unable to complete PROMs themselves.
**Use in clinical practice**
• Administering PROMs before the intake and at treatment evaluation moments provides valuable insights into the child’s perceived functioning and progress. This information can support goal setting, inform the clinical reasoning of the pediatric physical therapist,^9,10^ and facilitate shared-decision making.^11,12^
• When using the core PROM set in clinical practice it is highly important that the pediatric physical therapist discusses the PROM results with the patient to improve patient-healthcare professional communication, and to support willingness of patients to fill in PROMs.^9,10^
• To aid interpretation of PROM results for healthcare providers, children, and parents, these results can be presented in a visual or graphical format.^33^
**Use in research**
• In the context of research, the core PROM set can be used as a screener tool for eligibility criteria, and/or as outcomes of research.

These recommendations have been formulated by an expert panel, including experienced researchers in pediatric physical therapy, psychometrics, psychology and/or PRO(M)s.

CAT, computerized adaptive testing; PROs, patient-reported outcomes; PROM, patient-reported outcomes measures; PROMIS, patient-reported outcomes measurement information system.

## DISCUSSION

In this project, a multidisciplinary workgroup of experts in the fields of PPT, PRO(M)s, psychology, and psychometrics collaborated with pediatric physical therapists, adolescents in PPT, and their parents to create a generic core PRO(M) set for PPT. This core PRO(M) set can be implemented in daily care, research, and quality assessment for all children, aged 5–18 years, regardless of their medical indication within the field of PPT, to ensure comprehensive incorporation of physical, social, and mental well-being. The core PRO(M) set was designed to include a limited number of generic, age-appropriate PRO(M)s, to be relevant to most children in PPT and be minimally burdensome in application. Multiple frequently used PROMs with established psychometric properties, measuring the level of functioning, symptoms, or overarching health, have been incorporated in the core PRO(M) set.

The development of the core PRO(M) set for PPT demonstrates that many PROs are universally relevant to all children, regardless of their (medical) condition and age, as the included PROs reflect key generic outcomes identified in prior, non-PPT-specific PRO research.^18,25^ For example, the PROs in the core PRO(M) set for PPT are largely consistent with those included in the PROMIS Pediatric Core Domains^18^ and the GPROM set^6,25^ (a national recommendation for generic PROMs). This mirrors previous findings from content analyses of disease-specific PROMs, which have shown a consistent focus on a limited number of PROs relevant to most children.^6,16^ Together, these findings indicate that the identified PROs are largely generic, and that by aligning with existing initiatives, such as the Dutch GPROM set^24^ as PROMIS Pediatric Core Domains,^18^ international harmonization and comparability of outcomes can be achieved.

In our recommended PPT core PRO(M) set there is currently no suitable PROM for the PRO “participation.” As the topic of participation is a cornerstone of modern PPT,^8,9^ and was ranked as the most important PRO in our survey amongst pediatric physical therapists, it is concerning that no suitable PROM was found. One major challenge lies in the conceptual complexity of participation. Imms et al^34,35^ described participation as consisting of 2 core components: *attendance* (being there) and *involvement* (the subjective experience while attending). While some tools measure attendance, they often neglect involvement, including elements like motivation, social connection, and enjoyment.^34^ Attempts to include both dimensions often result in long instruments (Supplemental Digital Content 3, available at: https://links.lww.com/PPT/A698: eg, Participation and Environment Measure for Children and Youth [58 items], the Children’s Assessment of Participation and Enjoyment [55 items], Life Habits Questionnaire [Shortened version: 69 items]). Furthermore, the many participation domains, and individual variation in what are relevant domains, makes it difficult to design standardized measures that are both comprehensive and relevant across populations, and finally, the choice of an instrument may also depend on the purpose.^36^ Some individualized interview-based measures, such as the Canadian Occupational Performance Measure,^37^ aim to capture participation through an individualized approach. While such instruments allow for a more tailored and context-sensitive assessment, their use remains challenging as psychometric quality highly depends on how well topics are being addressed and scored.^38^ The Pediatric Spinal Cord Injury Measure Pediatric Measure of Participation is a promising pediatric PROM that assesses participation, available as short forms (≤12 items) or in CAT format (Supplemental Digital Content 3, available at: https://links.lww.com/PPT/A698).^39,40^ Currently, its response options are specific to children with spinal cord injury, but these could be easily adapted for broader use. However, psychometric properties would need to be evaluated in general and clinical populations after such adaptation. Future research should focus on the adaptation or development of a feasible generic PROM, sensitive to the nuanced nature of participation.

This paper offers several recommendations (Table 3) for sustainable implementation of the PPT core PRO(M) set in research and daily clinical care. These recommendations focus on (1) minimizing patient burden (eg, by using CATs when possible and using NRS questions as screener for administered longer PROMs), (2) allowing the patient to report on their own perceived experiences (eg, by allowing children to complete PROMs themselves, without the presence of a healthcare professional or researcher), (3) maintaining high PROM response rates (by ensuring PROMs are discussed during a PPT visit), (4) using PROMs to monitor treatment progress (by using them at evaluations), and (5) covering all relevant PROs in certain patient populations (by encouraging supplementing the core PROM set with disease-specific PROMs, however only when necessary). These strategies can be more easily integrated when PROMs are delivered through a digital platform, allowing patients to complete them via their own device from their home environment, and enabling healthcare providers to directly access responses.^41^ In the Netherlands, the PPT core PROM set, including the CAT versions of PROMs, is available through the personalized online KLIK PROM portal (www.klik-fysiotherapie.nl). However, real-life implementation still poses some challenges. Barriers and facilitators towards its implementation are currently investigated by our research group.

Our results must be interpreted within the limitations of the study. First, although adolescents’ and caregivers’ voices were heard in terms of what they deemed important PROs, they were not involved in the prioritization of it. Adolescents and caregivers were also not involved in the selection of the PROMs. Their involvement could be considered in future evaluations of the core PROM set. Nevertheless, many children participated in the cognitive debriefing of the PROMIS item banks, supporting the relevance and comprehensibility of these measures. Second, our qualitative study was conducted within the Dutch healthcare system, which may limit its international applicability. However, our scoping review, an additional key source for identifying relevant PROs, was not restricted to the Netherlands and included international literature. Additionally, since international core sets like the PROMIS Pediatric Core Domains^18^ yield similar relevant PRO, we believe this limitation is minimal. Third, the selection of PROMs was based on the GPROM set, a Dutch initiative, which may pose some limitations in terms of international applicability. However, as the recommended PROMs in this set are primarily based on PROMIS instruments, an international initiative, and are all available in multiple languages, this limitation is likely to be minimal. Fourth, in our PPT core PRO(M) set, we did not distinguish between different subdomains within the domain of participation. Our research focused exclusively on the broader domain of participation to align closely with the GPROM set (where no distinction between subdomains for “participation” are made), aiming to enhance the standardization of PROMs. However, this approach could be seen as a limitation of our study. Finally, evidence on the psychometric properties of PROMIS instruments for children using mobility aids remains limited. Further research is needed to assess their validity and reliability in this population.

## CONCLUSIONS

We present a carefully created core PRO(M) set for use in PPT daily care, research, and quality assessment. The core PRO(M) set stimulates standardization and harmonization in PROM use in PPT and provides guidance for healthcare professionals. Furthermore, we provide recommendations for the use of the core PRO(M) set, to ensure optimal benefit and implementation. Future research should focus on the development of a feasible generic participation PROM, sensitive to the nuanced nature of this construct, and the psychometric properties of PROMIS instruments for children using mobility aids.

## ACKNOWLEDGMENTS

We would like to thank Tessa van Gastel and Anouk Groenewegen for assisting with the focus groups, and all individuals and organizations that helped in recruiting participants for this study. We would like to thank the following pediatric physical therapy practices for hosting focus groups: PMC in Balans, DINK Kinderfysiotherapie, Fysiotherapie Nuenen, and Fysiotherapie Houwer en Ruijs. Above all, we want to thank all adolescents, parents, and pediatric physical therapists for participating in this study.

## Supplementary Material


