# Supplementary material for: Development of a Core Patient-Reported Outcome (Measures) Set for Pediatric Physical Therapy
Source: Pediatr Phys Ther. 2026 Jul 31;38(3):364–73. doi: 10.1097/PEP.0000000000001304 (PMC13432966; doi:10.1097/PEP.0000000000001304)
Supplement: Supplementary file 1 [file ppyty-38-364-s001.pdf]

Supplementary file 1: Models Integrated into the Standard Set of PROs by Oude Voshaar et al.

Models integrated into the standard set of PROs by Oude Voshaar et al.[1, 2]

This standard set is based on (1) the Alonso & Valderas model[3] (a classification system of PRO measures combining the Wilson and Cleary model[4] and the International Classification of Functioning, Disability and Health framework (ICF)[5]) and (2) the Patient-Reported Outcomes Measurement Information System® (PROMIS®) conceptual framework[6] (a guide for the development of distinct PROs about physical, mental, and social health). This standard set was selected as it is grounded in widely used frameworks across various healthcare domains (such as the ICF in PPT and rehabilitation, and the Wilson & Cleary model in psychology), and PRO-specific frameworks (Alonso & Valderas and the PROMIS conceptual model).

[1] Oude Voshaar M, Terwee CB, Haverman L, van der Kolk B, Harkes M, van Woerden CS, et al. Development of a standard set of PROs and generic PROMs for Dutch medical specialist care : Recommendations from the Outcome-Based Healthcare Program Working Group Generic PROMs. Qual Life Res. 2023;32:1595-605.
[2] van Silfhout NY, van Muilekom MM, van Karnebeek CD, Haverman L, van Eeghen AM. PROs for RARE: protocol for development of a core patient reported outcome set for individuals with genetic intellectual disability. Orphanet J Rare Dis. 2024;19:354.
[3] Valderas JM, Alonso J. Patient reported outcome measures: a model-based classification system for research and clinical practice. Qual Life Res. 2008;17:1125-35.
[4] Wilson IB, Cleary PD. Linking clinical variables with health-related quality of life. A conceptual model of patient outcomes. JAMA. 1995;273:59-65.
[5] Ustun TB, Chatterji S, Bickenbach J, Kostanjsek N, Schneider M. The International Classification of Functioning, Disability and Health: a new tool for understanding disability and health. Disabil Rehabil. 2003;25:565-71.
[6] Tucker CA, Cieza A Fau - Riley AW, Riley Aw Fau - Stucki G, Stucki G Fau - Lai JS, Lai Js Fau - Bedirhan Ustun T, Bedirhan Ustun T Fau - Kostanjsek N, et al. Concept analysis of the patient reported outcomes measurement information system (PROMIS(®)) and the international classification of functioning, disability and health (ICF).
