# Supplementary material for: Development of a Core Patient-Reported Outcome (Measures) Set for Pediatric Physical Therapy
Source: Pediatr Phys Ther. 2026 Jul 31;38(3):364–73. doi: 10.1097/PEP.0000000000001304 (PMC13432966; doi:10.1097/PEP.0000000000001304)
Supplement: Supplementary file 2 [file ppyty-38-364-s002.pdf]

Supplementary file 2. Operationalization and comprehensive assessment and of PROs

Table 1: PROs identified through focus groups and interviews. This table indicates which PROs were removed or merged in the various stages of the comprehensive assessment and operationalization of PROs (step 2b of the manuscript)

PRO	Removal of outcomes not deemed as important to measure as PRO in PPT according to the focus groups and interviews	Removal of non PROs	Removal of non-generic PROs	Merging of PROs falling under the same operationalization
Anger				
Anxiety				
Ball skills	■	■	■	■
Bowel and bladder control		■	■	■
Child- parent relation		■	■	■
Cognitive functioning				
Confidence				■
Depression				
Dizziness	■	■	■	■
Fatigue				
Fine motor skills/ upper extremity				
Functioning of family members		■	■	■
Goal setting		■	■	■
Gross motor skills/ mobility				
Lack of understanding from others	■	■	■	■
Motivation	■	■	■	■
Nausea	■	■	■	■
Pain				
Participation				
Perceived health				
Physical activity level		■	■	■
Physical fitness				■
Positive feelings	■	■	■	■
Quality of life				
Respiratory issues			■	■
Sensory integration		■	■	■
Sleep				
Social functioning				
Speech	■	■	■	■
Strength				■
To be able to set boundaries	■	■	■	■
Wanting to be normal				■

■ = PRO removed

□ = PRO moves to the next step

Table 2: PROs identified in literature review on PROs measured in pediatric physiotherapy (Korteling D.L., Limmen S., et al.). This table indicates which PROs were removed or merged in the various stages of the comprehensive assessment and operationalization of PROs (step 2b of the manuscript)

**PRO**	**Removal of non PROs**	**Removal of non-generic PROs**	**Merging of PROs falling under the same operationalization**
Activities and participation			
Anxiety			
Body image			
Community, social and civic life			
Confidence			
Defecation functions			
Depression			
Education			
Emotional functions			
Family relationships			
Fatigue			
Functions of the joints and bones			
Functions of the respiratory system			
Functions related to the digestive system			
General health perceptions			
General physical endurance			
General tasks and demands			
Global psychosocial functions			
Goal attainment			
Hand and arm use			
Intellectual functions			
Interpersonal interactions and relationships			
Managing one's own activity level			
Mental functions			
Mobility			
Motivation			
Neuromusculoskeletal and movement-related functions			
Orientation to self			
Overall quality of life			
Pain			
Perceptual functions			
Recreation and leisure			
Regulating behaviors within interactions			
Self-care			
Sensory functions			
Services, systems and policies			
Sleep functions			
Sports			
Treatment expectation			
Undertaking multiple tasks independently			

= PRO removed

= PRO moves to the next step

Table 3: Combined PROs from qualitative research (Table 1) and literature review (Table 2)

Overlapping PROs from qualitative research and literature review		
**Qualitative research**		**Literature review**
1	Anxiety	Anxiety
2	Cognitive functioning	Intellectual functions
3	Depression	Depression
4	Fatigue	Fatigue
5	Fine motor skills/ upper extremity	Hand and arm use
6	Gross motor skills/ mobility	Mobility
7	Pain	Pain
8	Participation	Activities and participation
9	Social functioning	Interpersonal interactions and relationships
10	Perceived health	General health perceptions
11	Quality of life	Overall quality of life
12	Sleep	Sleep functions
**Unique PROs from qualitative research and literature review**		
**Qualitative research**		**Literature review**
13	Anger	

Table 4: Operationalization of PROs based on the paediatric GPROM set and the ICF model

	Generic PRO		Operationalization	Subdomain
Overarching	Quality of life		Patient's assessment of their current quality of life	Summary judgment in a single item. The patient weighs all factors (including PROs) that determine their quality of life, which may include questions like:<br>- How happy are you with your life?
	Perceived health		Patient's assessment of their current perceived health	Summary judgment in a single item. The patient weighs all factors (including PROs) that determine their perceived health.
Functioning	Physical functioning/ activity		Ability to perform a task or activity	- Upper extremity functioning (including self-care)<br>- Gross motor functioning/ mobility
	Participation/ social functioning	Participation	Actual participation in social roles and activities	- Leisure activities<br>- Sport participation
		Social functioning	Ability to participate in social roles and activities	- Social skills to participate in social roles and activities<br>- Interaction with peers<br>(School absenteeism and family/home situation are asked via an additional question, not with a PROM)
	Mental functioning	Anxiety	Experienced complaints of fear, panic, worry, tension/stress, nervousness, and restlessness	None
		Depression	Experienced depressive symptoms	None
		Anger	Experienced feelings of anger	None
	Cognitive functioning		Paying attention/concentrating, quickly processing information, being flexible (accepting changing situations), remembering things.	None
Symptoms	Fatigue	Intensity	Degree (intensity) of fatigue	None
		Interference	Interference in daily life due to fatigue	None
	Pain	Intensity	Degree (intensity) of pain	None
		Interference	Interference in daily life due to pain	None
	Sleep		Experienced quality of sleep	- Falling asleep<br>- Staying asleep / quality of sleep
