# Supplementary material for: Development of a Core Patient-Reported Outcome (Measures) Set for Pediatric Physical Therapy
Source: Pediatr Phys Ther. 2026 Jul 31;38(3):364–73. doi: 10.1097/PEP.0000000000001304 (PMC13432966; doi:10.1097/PEP.0000000000001304)
Supplement: Supplementary file 3 [file ppyty-38-364-s003.pdf]

Supplementary file 3. PROM selection

Figure 1. Selection of suitable PROMs for the PPT core PROM set. *Other sources here were: the scoping review on PRO(M)s in PPT,[1] personal networks and www.meetinstrumentenzorg.nl

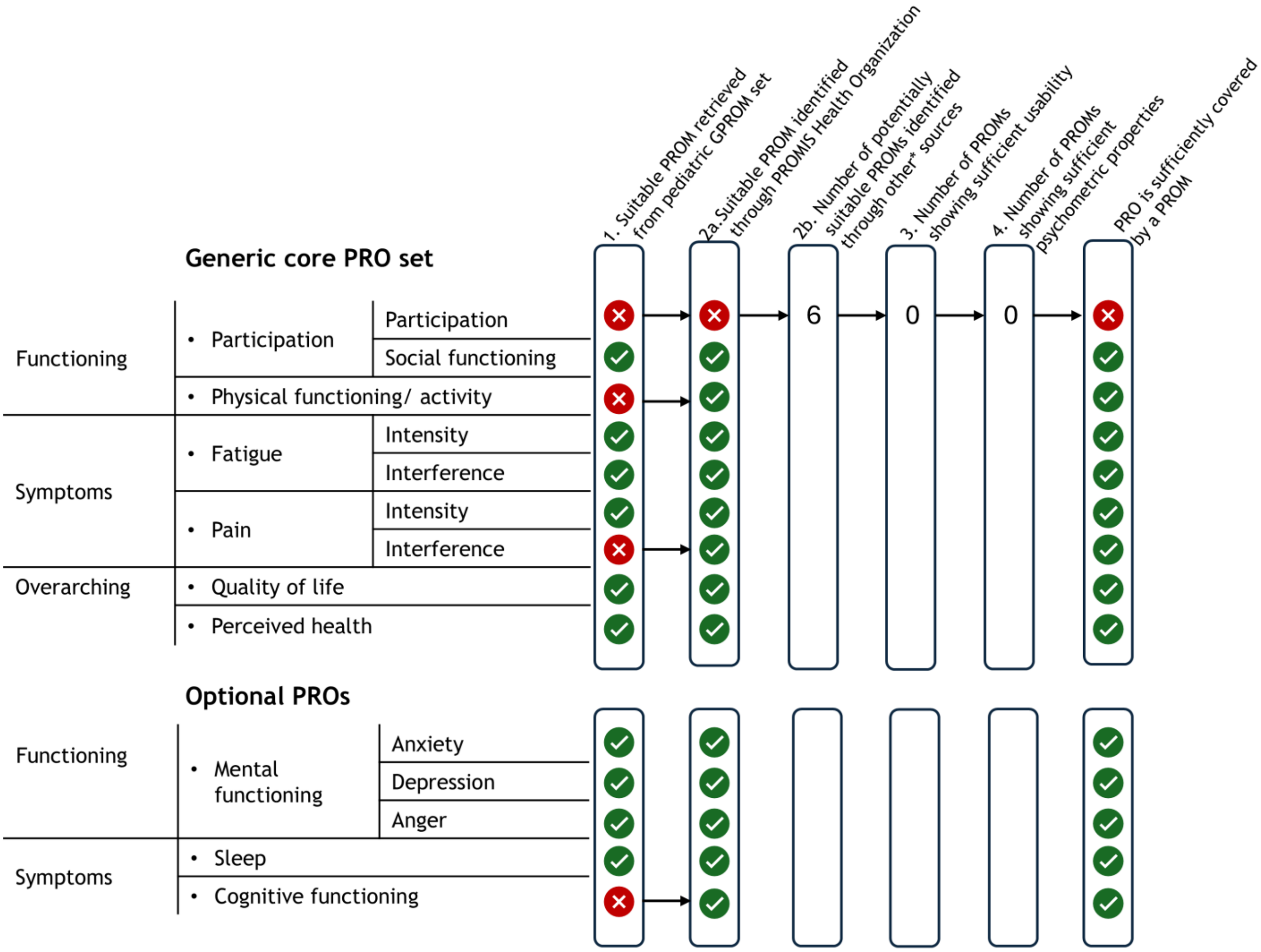

Table 1. Rating of usability of potential PROMs for the coreset of PROMs. For usability, we considered: 1) the comprehensiveness of the questions, 2) applicability across a wide age range (preferably 5-18 years), 3) a completion time of less than approximately 1 minute, 4) no associated costs, and 5) availability in multiple languages to support international use.

PROM	Comprehens-iveness	Age range	Number of items	Associated costs	Available in multiple languages to support international use
**Core PRO: Physical Functioning**					
PROMIS pediatric/parent-proxy Upper Extremity short-form[2]	No*	**5-18 years**	**4**	**No**	**Yes**
**Core PRO: Participation**					
PROMIS Ability to Participate in Social Roles and Activities[3]	*No*	*>18 years*	**4**	**No**	**Yes**
Participation and Environment Measure for Children and Youth[4]	**Yes**	**5-17 years**	*58*	*Yes*	**Yes**
Canadian Occupational Performance Measure[5]	**Yes**	**All ages**	*N.A. (interview)*	*Yes*	**Yes**
Children's Assessment of Participation and Enjoyment[6]	**Yes**	**6-21 years**	*55*	*Yes*	**Yes**
Life Habits Questionnaire (Shortened)[7]	*No*	**All ages**	*69*	**No**	**Yes**
Utrecht Scale for Evaluation of Rehabilitation (- Participation) Adolescents[8]	**Yes**	>12 years	*32*	**No**	**Yes**
Pediatric Spinal Cord Injury Measure (PEDI-SCI): Pediatric Measure of Participation (PMOP) short-form[9]	*No***	**8-21 years**	**≤12**	Probably not	**Yes**
**Core PRO: Pain Interference**					
PROMIS pediatric/parent-proxy Pain Interference short-form[2]	**Yes**	**5-18 years**	**4**	**No**	**Yes**
**Optional PRO: Cognitive Functioning**					
PROMIS pediatric/parent-proxy Cognitive Functioning short-form[10]	**Yes**	**5-18 years**	**4**	**No**	**Yes**

When criterion is met, this indicated in **green** and **bold**. When a criterion is not met, this is indicated in *red* and *italics*. When a criterion is not met, but might be sufficient, this is indicated in orange.

*The PROMIS pediatric/parent-proxy Upper Extremity short-form partially covers our operationalization of the PRO 'physical functioning', focusing solely on fine motor skills/upper extremity functioning while omitting mobility. However, when using this PROM in tandem with the PROMIS pediatric/parent-proxy Mobility short-form, we ensure full coverage of the PROs operationalization.

** The PEDI-SCI PMOP is not a generic PROM, as it specifically identifies whether respondents are unable to participate in an activity due to their spinal cord injury or disfunction. Therefore, this PROM is not comprehensive for all children receiving pediatric physical therapy.

Table 2. Rating of clinimetric properties of potential PROMs for the coreset of PROMs.

PROM	Content validity	Structural validity	Internal consistency	Cross-cultural validity (Dutch compared to U.S.)	Reliability	Responsiveness
**Core PRO: Physical Functioning**						
PROMIS pediatric/parent-proxy Upper Extremity short-form	√[2]	√[11]	√[11]	√[11]	√[11]	√[13]
**Core PRO: Pain Interference**						
PROMIS pediatric/parent-proxy Pain Interference short-form	√[2]	√[11]	√[11]	√[11]	√[11]	√[13]
**Optional PRO: Cognitive Functioning**						
PROMIS pediatric/parent-proxy Cognitive Functioning short-form	√[10]	Not studied (yet)	√[12]	Not studied (yet)	√[12]	√[13]

1. Korteling DL, Limmen S, Ketelaar M, et al. Patient Reported Outcomes in Pediatric Physical therapy: A Scoping Review and Evidence Map. (Submitted).
2. Irwin DE, Stucky BD, Thissen D, et al. Sampling plan and patient characteristics of the PROMIS pediatrics large-scale survey. *Qual Life Res*. May 2010;19(4):585–94. doi:10.1007/s11136-010-9618-4
3. Terwee CB, Crins MHP, Boers M, de Vet HCW, Roorda LD. Validation of two PROMIS item banks for measuring social participation in the Dutch general population. *Qual Life Res*. Jan 2019;28(1):211–220. doi:10.1007/s11136-018-1995-0
4. Coster W, Law M, Bedell G, Khetani M, Cousins M, Teplicky R. Development of the participation and environment measure for children and youth: conceptual basis. *Disabil Rehabil*. 2012;34(3):238–46. doi:10.3109/09638288.2011.603017
5. Law M, Baptiste S, McColl M, Opzoomer A, Polatajko H, Pollock N. The Canadian occupational performance measure: an outcome measure for occupational therapy. *Can J Occup Ther*. Apr 1990;57(2):82–7. doi:10.1177/000841749005700207
6. King GA, Law M, King S, et al. Measuring children's participation in recreation and leisure activities: construct validation of the CAPE and PAC. *Child Care Hlth Dev*. Jan 2007;33(1):28–39. doi:10.1111/j.1365-2214.2006.00613.x
7. Gelderblom GJ, de Witte LP, Noreau L, Fougeyrollas P, Vincent C. The LIFE-H: Assessment of the quality of social participation. *Technology and Disability*. 2002;14(3):113–118. doi:10.3233/tad-2002-14306
8. Post MW, van der Zee CH, Hennink J, Schafrat CG, Visser-Meily JM, van Berlekom SB. Validity of the utrecht scale for evaluation of rehabilitation-participation. *Disabil Rehabil*. 2012;34(6):478–85. doi:10.3109/09638288.2011.608148
9. Mulcahey M. Pediatric Measure of Participation (PMoP) User ManualVersion 1.0. Philadelphia, PA Thomas Jefferson University; 2020.
10. Lai JS, Butt Z, Zelko F, et al. Development of a parent-report cognitive function item bank using item response theory and exploration of its clinical utility in computerized adaptive testing. *J Pediatr Psychol*. Aug 2011;36(7):766–79. doi:10.1093/jpepsy/jsr005
11. Korteling DL, Ketelaar M, Limmen S, et al. Psychometric properties and reference values of the Patient-Reported Outcomes Measurement Information System (PROMIS®) pediatric item banks Mobility, Upper Extremity and Pain Interference in the Dutch population. (Submitted).
12. Becker H, Stuifbergen A, Lee H, Kullberg V. Reliability and Validity of PROMIS Cognitive Abilities and Cognitive Concerns Scales Among People with Multiple Sclerosis. *Int J MS Care*. Spring 2014;16(1):1–8. doi:10.7224/1537-2073.2012-047
13. Hinds PS, Wang J, Cheng YI, et al. PROMIS pediatric measures validated in a longitudinal study design in pediatric oncology. *Pediatr Blood Cancer*. May 2019;66(5):e27606. doi:10.1002/pbc.27606
